# Unscrambling the Provenance of Eggs by Combining Chemometrics and Near-Infrared Reflectance Spectroscopy

**DOI:** 10.3390/s22134988

**Published:** 2022-07-01

**Authors:** Louwrens Christiaan Hoffman, Dongdong Ni, Buddhi Dayananda, N Abdul Ghafar, Daniel Cozzolino

**Affiliations:** 1 Queensland Alliance for Agriculture and Food Innovation, Centre for Nutrition and Food Sciences, The University of Queensland, St. Lucia, QLD 4072, Australia; louwrens.hoffman@uq.edu.au (L.C.H.); d.ni@uq.edu.au (D.N.); 2 School of Agriculture and Food Sciences, The University of Queensland, St. Lucia, QLD 4072, Australia; b.dayananda@uq.edu.au (B.D.); n.abdulghafar@uq.net.au (N.A.G.)

**Keywords:** eggs, albumin, yolk, NIR, linear discriminant analysis

## Abstract

Issues related to food authenticity, traceability, and fraud have increased in recent decades as a consequence of the deliberate and intentional substitution, addition, tampering, or misrepresentation of food ingredients, where false or misleading statements are made about a product for economic gains. This study aimed to evaluate the ability of a portable NIR instrument to classify egg samples sourced from different provenances or production systems (e.g., cage and free-range) in Australia. Whole egg samples (n: 100) were purchased from local supermarkets where the label in each of the packages was used as identification of the layers’ feeding system as per the Australian legislation and standards. The spectra of the albumin and yolk were collected using a portable NIR spectrophotometer (950–1600 nm). Principal component analysis (PCA) and linear discriminant analysis (LDA) were used to analyze the NIR data. The results obtained in this study showed how the combination of chemometrics and NIR spectroscopy allowed for the classification of egg albumin and yolk samples according to the system of production (cage and free range). The proposed method is simple, fast, environmentally friendly and avoids laborious sample pre-treatment, and is expected to become an alternative to commonly used techniques for egg quality assessment.

## 1. Introduction

Issues related to food authenticity, traceability, and fraud have increased in recent decades because of the deliberate and intentional substitution, addition, tampering, or misrepresentation of food ingredients, where false or misleading statements are made about a product for economic gains [1,2]. Thus, the need has increased for reliable analytical methods to monitor and test both authenticity and fraud in food ingredients and products [1,2,3,4,5]. 

The term food fraud is associated with the selling of a cheap food ingredient or product at the price of an expensive one [2,5,6,7]. Food fraud has been a common challenge since ancient times due to reasons of profitability, and to mask the unusual appearance or taste of perishable foods [2,5,6,7]. In recent decades, numerous fraudulent practices have been identified, and monitoring using modern analytical techniques and instrumentation has been developed [2,5,6,7]. Yet, the growth in global food supply chains, and the incidence and effects of food fraud, have increased in recent years in several countries [2,6,7]. Similarly, several analytical techniques (e.g., DNA, chromatographic, and spectroscopy techniques) have been developed and utilized as tools to detect issues associated with fraud along the food supply and value chains [2,6,7,8,9].

Eggs are an important staple food in human diets, particularly for their high nutritious value due to their protein, vitamins, omega-3 fatty acids, lutein, and selenium content [10]. Due to changes in consumer preferences, and the high demand for high-quality and nutritious foods, the consumption and demand for eggs produced under more environmentally and animal welfare-friendly conditions have increased [5,8,9,11]. The egg is regarded as one of the least expensive sources of animal protein [12]. As a consequence, fraud involving the false declaration of information related to egg quality has often been reported, including substitution or mislabeling [13]. Pandemics (e.g., COVID-19), conflicts between countries, and climate change are significant disruptions in food supply chains, contributing to interrupting or slowing food trade, in addition to increasing food demand and lifting prices [14]. As highlighted by different scholars, these scenarios have created favorable conditions for food fraud, even in low-cost products, such as eggs [1,2,11]. In this context, the determination of authenticity and provenance, and their effects on egg consumption at the various stages of the supply chain (e.g., farm, retailers, supermarket), by the utilization of reliable, fast, and cost-effective technology is of paramount importance for the industry [1,2,3,11].

As discussed above, several analytical techniques have been tested and are available to evaluate and monitor food authenticity, provenance, and fraud issues [2,3]. In particular, applications based on the utilization of vibrational spectroscopy (e.g., mid- and near infrared, Raman spectroscopy) have been developed in past decades due to the many advantages of these techniques when compared with traditional routine methods of analysis (e.g., chromatographic and wet chemistry such as proximate analysis) [2]. These advantages include non-destruction of the sample, minimal or no sample preparation, and no requirement for the use of hazardous chemicals during the analysis (green technology) [2,15,16,17]. In addition, one characteristic of these techniques is the time required during the analysis of a given sample; that is, only a few seconds or minutes are needed for data collection (e.g., scanning or spectra collection) [2,15,16,17].

Near-infrared (NIR) spectroscopy has been utilized to determine egg composition and quality, in which freshness has been one of the parameters related to quality most reported by different authors [5,18,19,20,21,22,23,24,25,26,27,28,29,30,31]. Diffuse reflectance Fourier transform near-infrared (FT-NIR) spectroscopy was reported to predict the thickness of the egg white (i.e., height), with a determination coefficient of 0.82 [29]. Egg freshness measured as HU (Haugh units, index of freshness) were predicted by combining NIR spectroscopy, artificial neural networks (ANNs), and genetic algorithms (GAs) (correlation coefficient of 0.88) [22]. The utilization of visible (VIS) and NIR transmission spectroscopy was also reported to predict egg freshness [30,31]. Yet, no studies have evaluated NIR as a tool for verification of egg provenance or the system of production.

The objective of this study was to evaluate the ability of a portable NIR instrument to classify egg white and yolk samples collected from eggs sourced from different provenances or production systems (e.g., free range and cage) in Australia.

## 2. Materials and Methods

### 2.1. Samples

A total of 100 whole and unfertilized egg samples were purchased from local supermarkets where the label in each of the boxes or package was used as the identification of the feeding system employed to feed the layer hens. The production systems used in this study were defined as cage and free-range. The definition of these systems is regulated by different organizations such as Eggs Standards of Australia, Australian Eggs, and Eggs Farmers of Australia. Fresh egg samples were broken on a Petri dish, then the egg white and yolk were scanned separately. During the process, we noticed that, in some samples, the egg white and yolk were mixed. These samples were not included in the classification. 

### 2.2. Near Infrared Data Collection

A portable NIR spectrophotometer (Micro-NIR 1700, Viavi, Milpitas, CA, USA) was used to collect the spectra of egg white and yolk (yellow) samples in the wavelength range between 950 and 1600 nm. The spectral resolution used in this study was 10 nm with no moving parts (Viavi Solutions, 2015, Milipitas, CA, USA). The instrument control and the acquisition of the diffuse reflectance spectra of the samples was achieved using proprietary software (Viavi Solutions, 2015, Milipitas, CA, USA). Prior to the scanning of the samples, the reflectance spectra of a white ceramic disk (Spectralon^®^) were collected, followed by a dark spectrum, as recommended by the instrument manufacturer. This process was repeated every 20 samples. The samples (egg white and yolk) were scanned using a Petri dish where the head of the sensor was moved to collect either the egg white or yolk spectra, respectively. 

### 2.3. Data Analysis and Classification

Prior to the data interpretation and chemometric analysis, the NIR data were transformed using the Savitzky–Golay second derivative (21 smoothing points and second polynomial order) [32,33]. Principal component analysis (PCA) and linear discriminant analysis (LDA) were used to analyze and interpret any trends in the data set and to develop a classification model to monitor the origin of the egg samples analyzed (The Unscrambler X, CAMO Analytics AS, Oslo, Norway). The NIR data and the information provided in the label of the egg carton or package were used to develop LDA classification models using the combination of egg white and yolk NIR data, the egg white NIR data, or the yolk NIR data. Full cross validation (leave one out) was used to develop and validate both the PCA and LDA models [32,34]. The proportion (in percentage terms) of correct, incorrect, and overall classification, the sensitivity, and the specificity were used to evaluate the LDA models developed.

## 3. Results and Discussion

The egg white accounts for almost two-thirds of the egg liquid weight and is composed of approximately 10% protein, 0.9% carbohydrates, and 0.5% ash [35,36]. The yolk contains most of the lipids (approx. 62% triglycerides, 33% phospholipids, and less than 5% cholesterol) of the egg and has slightly less than half of the egg proteins; the egg water content represents between 75 and 85% of the whole egg composition [35,36]. 

**Figure 1:**
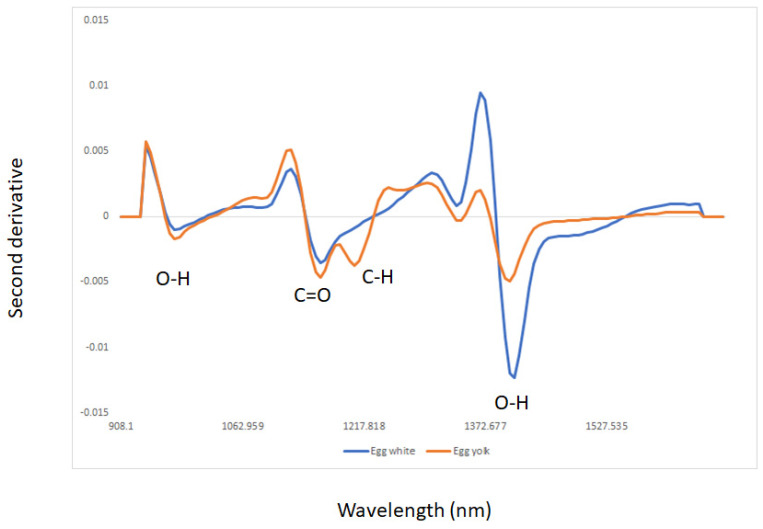
Average of the second derivative of egg white and yolk samples analyzed using near infrared reflectance spectroscopy.

**Figure 2:**
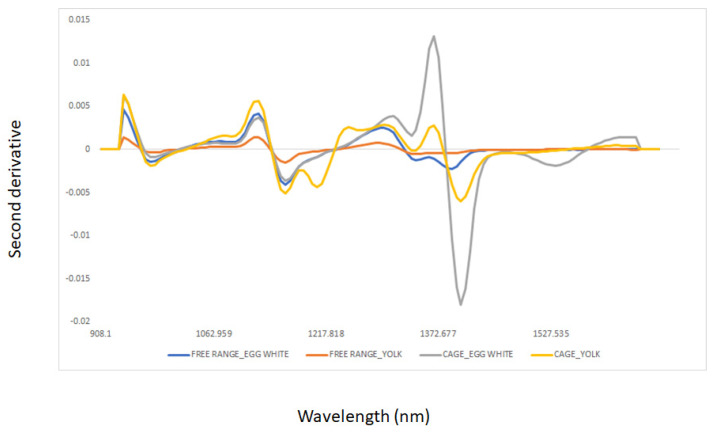
Average of the second derivative of egg white and yolk samples sourced from two production systems (cage and free range) and analyzed using near infrared reflectance spectroscopy.

The average second derivative of the NIR spectra of the egg white and yolk samples is shown in Figure 1. The samples analyzed showed main absorbances at the following wavelengths: 976 nm (O-H overtones), 1162 nm (C=O), 1205 nm (C-H and C-H_2_), 1342 nm (C-H_2_ and C-H_3_), and around 1405 nm (O-H overtones) [37]. Absorbance at 1162 nm may be associated with the absorption of lipids and fatty acids containing cis double bonds (second overtones C-H) (oleic acid), as reported by other researchers analyzing egg samples by NIR spectroscopy [38]. The absorption band around 1342 nm may be associated with the second aromatic C–H elongation overtone, mainly related to CH_2_ and CH_3_ from the saturated fatty acids present in the egg yolk [10,37]. The absorbance at 1405 nm may be associated with O-H (water), N-H (aromatic amines), and C-H combination tones [37]. The egg yolk samples showed higher absorbances than the egg white samples, having a distinctive absorption band at 1205 nm (C-H stretching second overtone of CH_2_ and CH) associated with carbohydrates and lipids, whereas the egg white samples showed a high absorbance band around 1430 nm mainly associated with water [37]. Figure 2 shows the average second derivative NIR spectra of egg white and yolk samples sourced from the two production systems, namely, cage and free range. Similar wavelengths as those described in the previous section can be observed in Figure 2. Differences were observed between cage and free-range egg samples around 1400 nm (O-H) associated with water content [10,37,38]. In addition to water, absorbances around 1162 nm (C=O and C-H) and 1205 nm (C-H and C-H_2_) associated with lipids and proteins were also observed. The average NIR spectra of the egg white samples sourced from the cage production system showed a characteristic band around 1205 nm mainly associated with lipids [10,37,38]. Principal component analysis (PCA) was used to visualize trends or groups in the dataset associated with the egg components (egg white and yolk) and production systems, and to identify unusual (outlier) samples [32,34].

**Figure 3:**
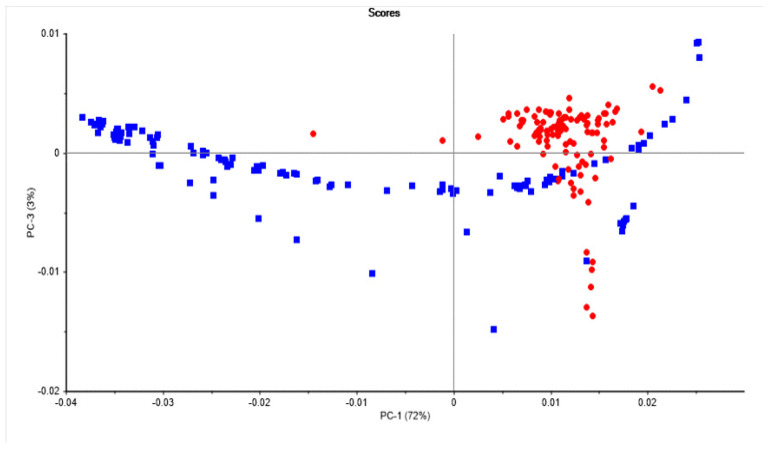
Principal component score plot of the egg white (blue squares) and yolk (red dots) samples analyzed using near-infrared reflectance spectroscopy.

**Figure 4:**
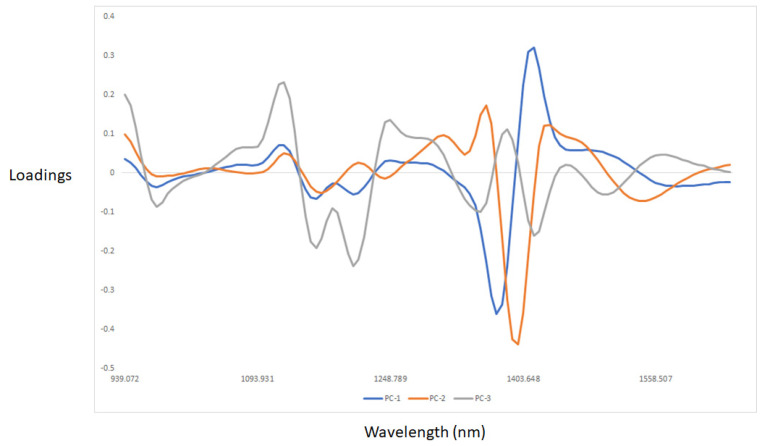
Loadings derived from the PCA analysis of the egg white and yolk samples analyzed using near-infrared reflectance spectroscopy.

Figure 3 and Figure 4 show the PCA score plot and loadings of the egg samples (egg white and yolk) analyzed using NIR spectroscopy. Principal components 1 and 3 are plotted in Figure 3. It was observed that the first three principal components explained 98% of the total variability in the NIR spectra of the egg samples evaluated. Plotting PC1 (72%) vs. PC3 (3%) showed a separation between the egg white and yolk samples. The egg white samples tend to scatter along PC1, whereas the egg yolk samples clustered together. Some of the samples analyzed were also overlapped, indicating the presence of outlier samples due to the mixing of the egg yolk with the egg white during the scanning. The highest loadings (see Figure 3) in PC1 were observed around 1400 nm, and were mainly driven by the absorption of O-H associated with water [36]. The first PC (72%) contributes to explaining the separation between the egg white samples. This can be attributed to small differences in water associated with egg freshness, as observed in Figure 2 and reported by other authors [24,25,26,27,28,29,30,39]. Similar trends have been also reported by other authors using bench NIR instruments [24,25,26,27,28,29,30,40,41]. Differences in PC1 can be also associated with the loss of moisture through the pores of the shell, and the structural change in proteins during storage [10]. The third PC (PC3, 3%) contributed to explaining the differences between egg white and yolk samples. The highest loadings in PC3 were observed at 1162 and 1205 nm (C-H and C-H_3_ bonds, respectively), 1347, 1422 (O-H bonds), and 1502 nm [37]. Overall, the loadings indicated that absorption bands associated with protein, water, fatty acids, and aromatic-like compounds were important and used by the model to classify the egg samples according to the production system. 

The discrimination results using the NIR data of all egg white and yolk combined, egg white, or yolk samples according to the feed system, combined with LDA, are reported in Table 1 (confusion matrix). The percentages of correct classifications obtained to differentiate free range from cage egg samples were 76, 86, and 86% using all samples (combining egg white and yolk), egg white, and yolk samples, respectively. The best classification results were achieved for free range egg samples, where 86, 92, and 89% of the samples were correctly classified using all, or egg white, or yolk, respectively. The differences in the classification rates obtained in this study were mainly related to composition, associated with both water and lipid content, as shown in the NIR spectra of the samples (see Figure 1 and Figure 2). As observed in Figure 2, egg samples sourced from cage production systems have more water than those from free range systems. Similar results for the classification of whole “natural” and commercial eggs [40], and the discrimination between free-range and cage yolk samples using a combination of VIS and NIR spectroscopy techniques and chemometrics, were reported by other authors [5,11]. These researchers evaluated egg yolk-filtrated samples from different origins using a combination of UV-VIS-NIR spectroscopy, indicating that the NIR spectra of the samples were able to classify egg samples according to organic, free range, barn, and cage systems [5,11].

## 4. Conclusions

The results obtained in this study show that the combination of chemometrics and NIR spectroscopy allowed for the classification of egg white and yolk samples according to the system of production (cage and free range). The proposed method is simple, fast, environmentally friendly, and avoids laborious sample pre-treatment, and is expected to become an alternative technique for egg quality assessment. Although the results of the present study are promising, further research is still needed to validate the existing classification models using an independent set of samples, and to evaluate the inclusion of other parameters associated with quality, such as chemical composition (e.g., protein and fat content) or shelf life, which may influence the classification results reported in this study.

## Figures and Tables

**Table 1:** Linear discriminant analysis results for the classification of the origin of eggs using all samples (combining egg white and yolk), egg white, or yolk, analyzed using near infrared spectroscopy.

Data Set	Origin	%CC	%IC	%OVCC	Sn	Sp
ALL (egg white and yolk combined)	Free range	86.8%	13.2%	76%	61%	50%
	Cage	49%	51%			
Egg white	Free range	92.8%	7.2%	86%	85.3%	68%
	Cage	67%	33%			
Egg yolk	Free range	89% (80/90)	11% (10/90)	86%	85.8%	70.1%
	Cage	74% (17/23)	26% (6/23)			

%CC: percentage of correct classification; %IC: percentage of incorrect classification; %OVCC: percentage of overall correct classification; Sn: sensitivity; Sp: specificity.

## Data Availability

Not applicable.
